# Alginate/Pluronic F127-based encapsulation supports viability and functionality of human dental pulp stem cell-derived insulin-producing cells

**DOI:** 10.1186/s13036-020-00246-1

**Published:** 2020-08-24

**Authors:** Suryo Kuncorojakti, Watchareewan Rodprasert, Supansa Yodmuang, Thanaphum Osathanon, Prasit Pavasant, Sayamon Srisuwatanasagul, Chenphop Sawangmake

**Affiliations:** 1 International Graduate Course in Veterinary Science and Technology, Faculty of Veterinary Science, Chulalongkorn University, Bangkok, 10330 Thailand; 2 Veterinary Stem Cell and Bioengineering Innovation Center (VSCBIC), Veterinary Pharmacology and Stem Cell Research Laboratory, Faculty of Veterinary Science, Chulalongkorn University, Bangkok, 10330 Thailand; 3 Research Affairs, Faculty of Medicine, Chulalongkorn University, Bangkok, 10330 Thailand; 4 Excellence Center for Advanced Therapy Medicinal Products, King Chulalongkorn Memorial Hospital, Bangkok, 10330 Thailand; 5 Department of Anatomy, Faculty of Dentistry, Chulalongkorn University, Bangkok, 10330 Thailand; 6 Center of Excellence in Regenerative Dentistry, Faculty of Dentistry, Chulalongkorn University, Bangkok, 10330 Thailand; 7 Department of Veterinary Anatomy, Faculty of Veterinary Science, Chulalongkorn University, Bangkok, 10330 Thailand; 8 Veterinary Clinical Stem Cell and Bioengineering Research Unit, Faculty of Veterinary Science, Chulalongkorn University, Bangkok, 10330 Thailand; 9 Department of Pharmacology, Faculty of Veterinary Science, Chulalongkorn University, Bangkok, 10330 Thailand

**Keywords:** Insulin-producing cells (IPCs), Dental pulp stem cells (DPSCs), Diabetes mellitus, Encapsulation, Alginate, Pluronic F127

## Abstract

**Background:**

Current approach for diabetes treatment remained several adverse events varied from gastrointestinal to life-threatening symptoms. Regenerative therapy regarding Edmonton protocol has been facing serious limitations involving protocol efficiency and safety. This led to the study for alternative insulin-producing cell (IPC) resource and transplantation platform. In this study, evaluation of encapsulated human dental pulp-derived stem cell (hDPSC)-derived IPCs by alginate (ALG) and pluronic F127-coated alginate (ALGPA) was performed.

**Results:**

The results showed that ALG and ALGPA preserved hDPSC viability and allowed glucose and insulin diffusion in and out. ALG and ALGPA-encapsulated hDPSC-derived IPCs maintained viability for at least 336 h and sustained pancreatic endoderm marker *(NGN3*), pancreatic islet markers (*NKX6.1, MAF-A, ISL-1, GLUT-2* and *INSULIN*), and intracellular pro-insulin and insulin expressions for at least 14 days. Functional analysis revealed a glucose-responsive C-peptide secretion of ALG- and ALGPA-encapsulated hDPSC-derived IPCs at 14 days post-encapsulation.

**Conclusion:**

ALG and ALGPA encapsulations efficiently preserved the viability and functionality of hDPSC-derived IPCs in vitro and could be the potential transplantation platform for further clinical application.

## Background

Diabetes mellitus is an intractable metabolic disease. Epidemiological studies covering 2.7 million of participants during 1980–2008 reported the global increases of glycemia and diabetes prevalence which were correlated with population growth and ageing [1]. Moreover, the global prevalence of diabetes is expected to be 693 million in all age-group worldwide by 2045 [2]. Several approaches have been clinically introduced to manage hyperglycemic conditions and consequence complications i.e. exogenous insulin and pharmacotherapeutic preparations [3]. However, there were some reports suggested an evidence of hypoglycemia and adverse events [4]. From these reasons, many of researchers have been trying in finding novel approach to cope diabetes and its complications. In 2000, the first human islet transplantation according to Edmonton protocol has been proposed [5]. However, there were some limitations i.e. adverse events of immunosuppressants, limited availability of donors, and limited duration of insulin independent period [6]. By the limitations of Edmonton protocol, trend of stem cell-based therapy has been announced as a candidate and promising protocol for diabetes treatment.

Various types of pluripotent stem cells have been employed for generation of insulin-producing cells (IPCs) in vitro. Derivation from mouse and human embryonic stem cells (ESCs) has been reported [7, 8], unfortunately, the ethical and tumorigenicity issue might hamper for the clinical application [9]. To address this problem, current plethora of studies are mainly focused on mesenchymal stem cells (MSCs) [10]. Various types of MSCs have been used for generation of IPCs in vitro i.e. human and mouse bone marrow-derived MSCs (BM-MSCs) [11]. Dental tissue-derived MSCs also can be proposed as an alternative source, in vitro generation of IPCs was firstly achieved by using MSCs isolated from SHED or stem cell from human exfoliated deciduous teeth [12]. Another study also reported that the human dental pulp stem cells (hDPSCs) had a good potency for in vitro differentiation toward IPCs [13]. Besides, hDPSCs have been proposed as the alternative stem cell resource due to their accessibility and availability [14, 15]. Furthermore, the advantages of hDPSC have been reported by several investigators i.e. the multilineage differentiation potential, the capacity for autologous and allogenic transplantation [16], the abundance in source and less ethical issue both in research and clinical translation [17].

Another challenge of cell-based therapy for translational study and clinical application is how to maintain the viability and functionality of the IPCs. Encapsulation can be applied to address this challenge due to the ability on rejection avoiding [18, 19]. Alginate is a material that widely used in biomedical field, drug delivery and tissue engineering [20], since it provides advantages i.e. less difficulties for application, effective immunobarrier activity [21] and cell viability support [20]. To avoid the cell protrusion that can lead rejection, multilayer encapsulation can be applied [22]. Pluronic F127, a synthetic and thermosensitive polymer can be used as a coating material of alginate due to their special characteristic i.e. showing non-toxicity, enhancing cell adhesion, supporting collagen production and promoting angiogenesis [23].

Currently, the application of multilayer encapsulation that incorporates alginate and pluronic F127 for stem cell-based diabetes therapy is not well established. This led to the pitfall of knowledge and opportunity for treating intractable disease. Therefore, this study is aimed to establish and validate the suitable encapsulation platform for IPCs in vitro*,* by using the hDPSC-derived IPCs. This knowledge will support the success of in vivo transplantation study which is critical for further clinical protocol establishment.

## Results

### Alginate/pluronic F127-based encapsulation supports viability of hDPSCs

**Fig. 1:**
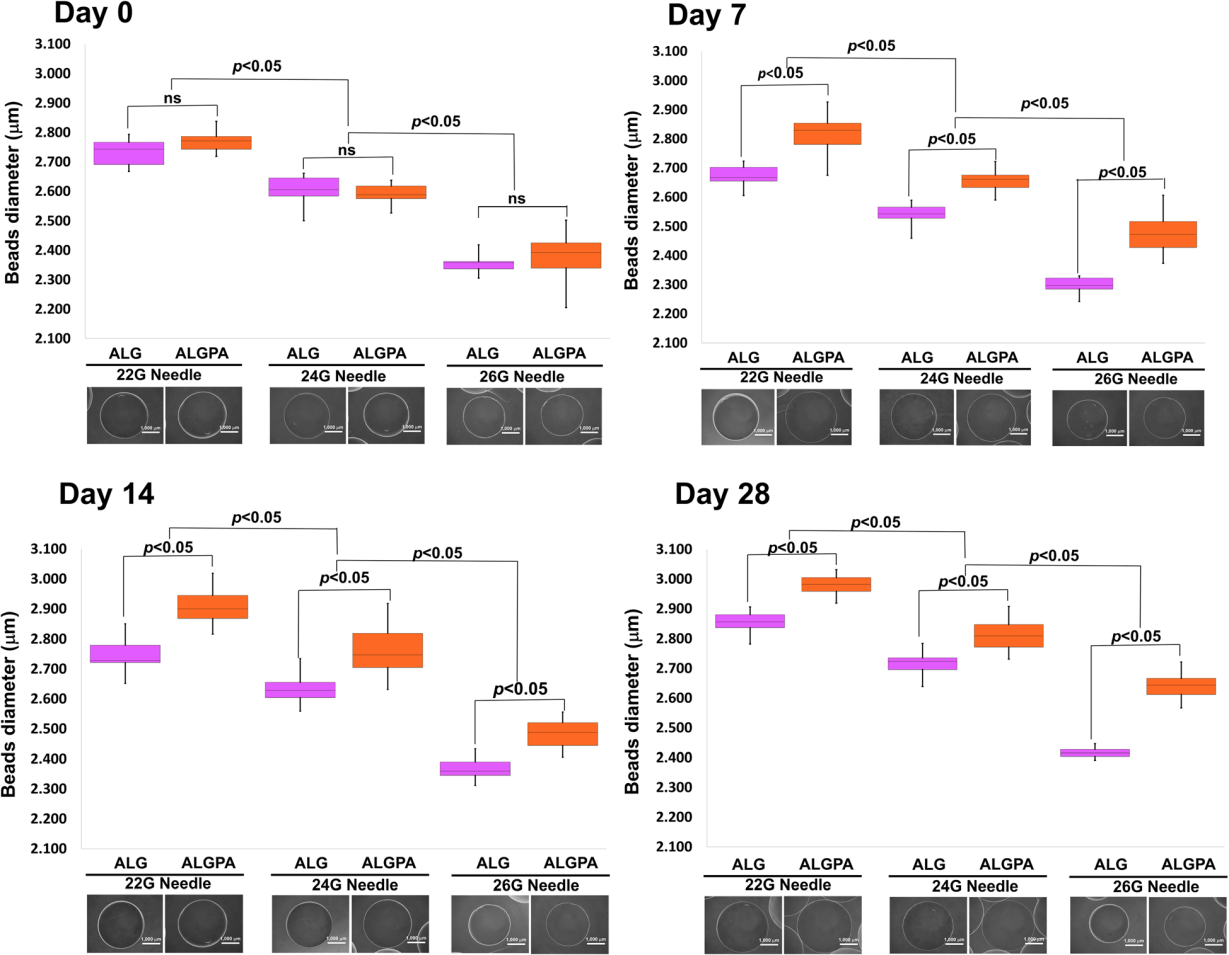
Bead diameter and morphology evaluation of ALG and ALGPA. Bead diameters and morphological appearances of ALG and ALGPA generated by manual extrusion through different sizes of needle (22G, 24G and 26G) at day 0, 7, 14 and 28 were illustrated. Bars indicated statistical relationship, *p* < 0.05 or not significant (ns)

For serving the IPC encapsulation, the diameter of needle plays an important role. Figure 1 visualized the effect of different size of needle (22G, 24G and 26G) on the diameter of the beads. The measurement of diameter was assessed at day 0, 7, 14 and 28. As shown in Fig. 1, manual extruding protocol using different needle size resulted in different size of beads diameter. At day 0 there is no significant difference on beads diameter produced from all different size of needle between alginate (ALG) and pluronic F127 coated alginate (ALGPA) encapsulation. The 22G needle resulted in the highest diameter in both encapsulation platform, respectively 2732.27 ± 44.62 mm and 2770.16 ± 34.08 mm for ALG and ALGPA, while 26G resulted in the lowest diameter size, 2355.22 ± 32.41 mm and 2379.70 ± 70.44 mm for ALG and ALGPA. Similar trend was also shown in day 7, 14 and 28 but significant differences of diameter were noted between ALG and ALGPA encapsulation. In the end of experiment the percentage swelling of both encapsulations were 2.61–4.50% and 7.64–10.91% for ALG and ALGPA respectively. The swelling percentage was assessed by calculating the percentage of final diameter at day 28 minus initial diameter at day 0, then compared to the baseline (diameter at day 0).

**Fig. 2:**
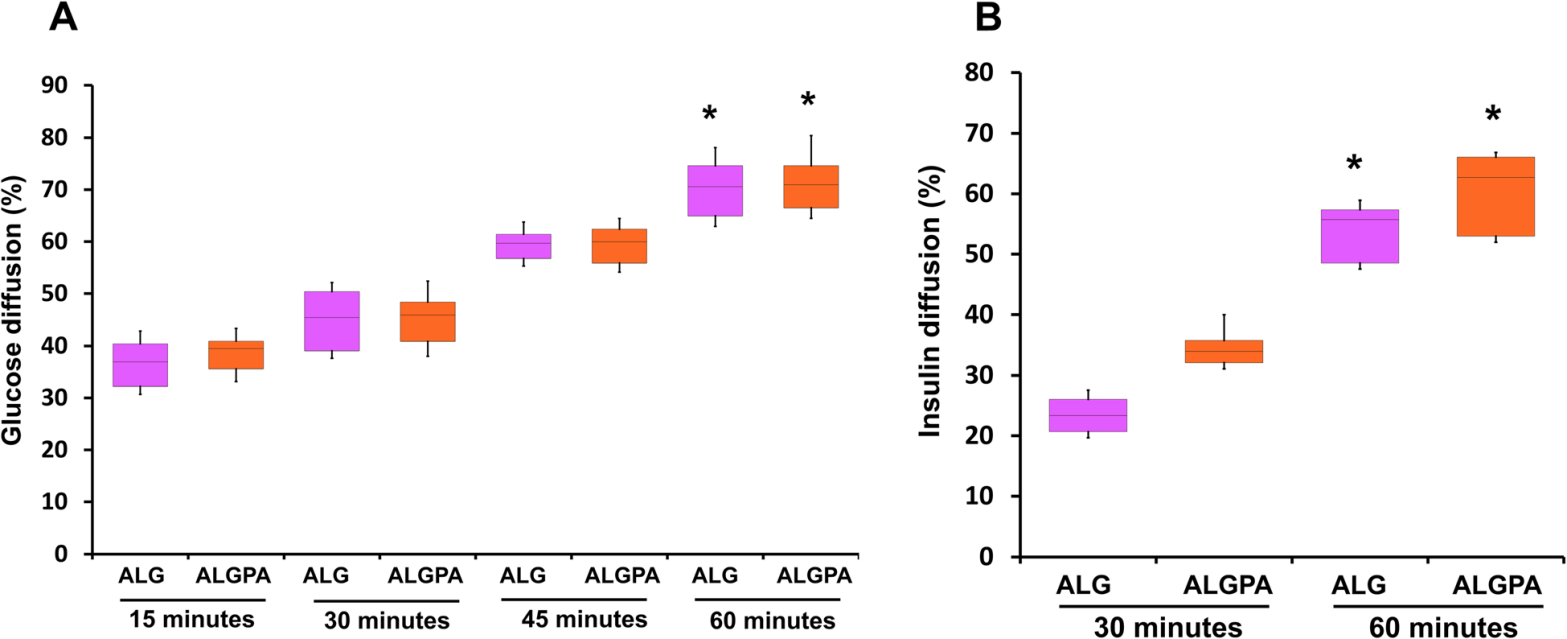
Glucose and insulin diffusion efficiency of ALG and ALGPA. The percentages of glucose diffusion **a** and insulin diffusion **b** across ALG and ALGPA as determined by modified trans-well diffusion assay were illustrated. The asterisks indicated significant difference, comparing with the initial condition at 15 min (glucose diffusion) and 30 min (insulin diffusion) (*p* < 0.05)

To evaluate diffusion ability of ALG and ALGPA, modified transwell diffusion assay was performed. Figure 2a showed the glucose diffusion in both encapsulation platform, after 60 min incubation. The percentage of glucose diffusion were 62.90–76.10% and 64.50–78.40% for ALG and ALGPA respectively. These results were significantly different compared to the baseline at 15 min. As shown in Fig. 2b, the percentage of insulin diffusion in ALG and ALGPA encapsulation after 60 min of incubation were 44.00–54.40% and 48.00–61.90% respectively which was significantly different (*p* < 0.05).

**Fig. 3:**
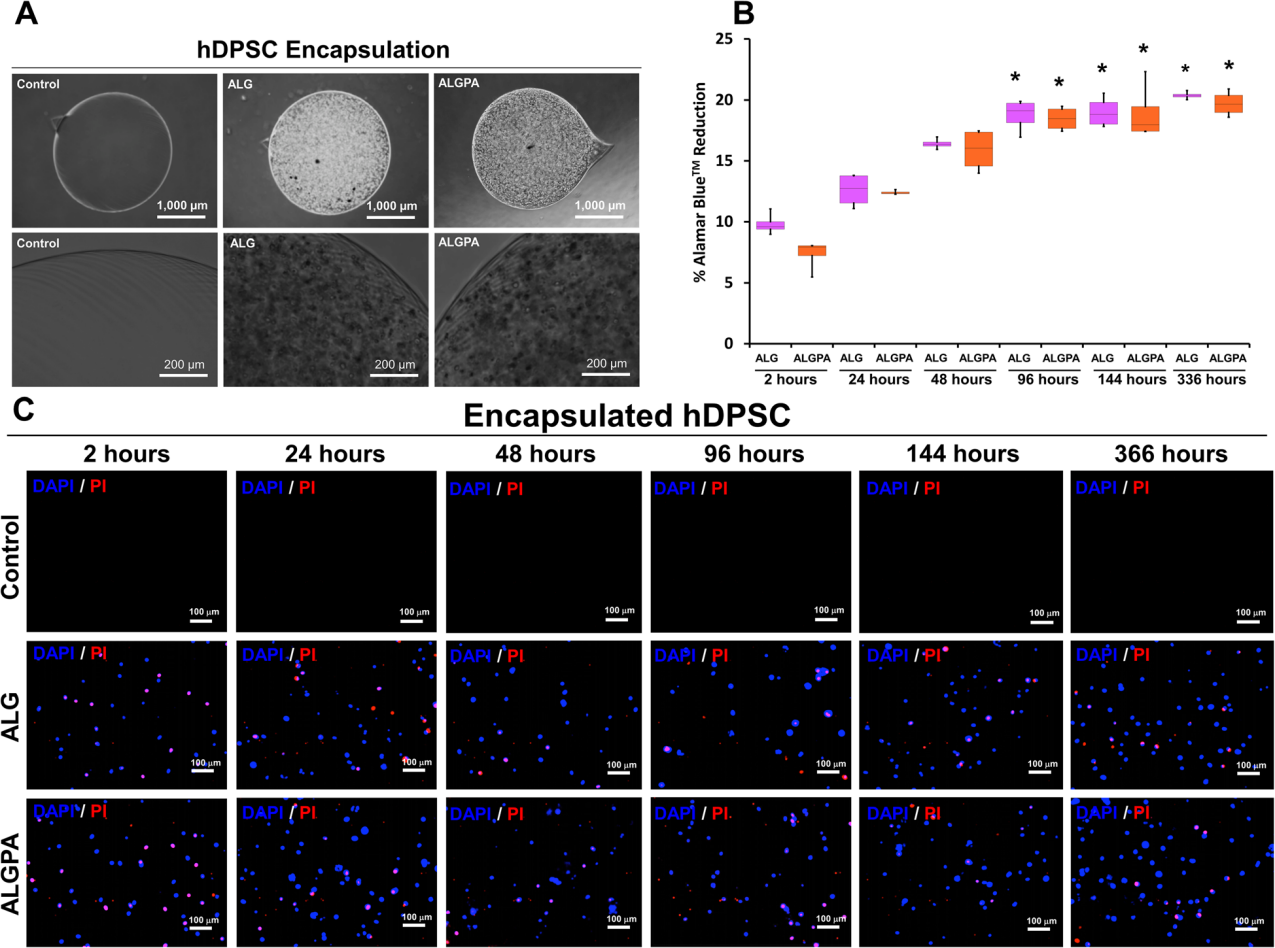
Morphology and viability evaluation of encapsulated hDPSCs using ALG and ALGPA. ALG- and ALGPA-encapsulated hDPSC morphologies were evaluated under light microscope **a**. The viability of encapsulated hDPSCs was also determined by alamarBlue™ assay **b** and live/dead staining **c**. The asterisks indicated significant difference, comparing with initial condition at 2 h (*p* < 0.05)

Visualization of encapsulated hDPSC morphology under inverted microscope showed that the hDPSCs were dispersed into single cells inside the alginate capsule (Fig. 3a**)**. Viability assessment of post-encapsulated hDPSCs was performed by quantitative method of alamarBlue™ assay and live/dead staining. As metabolic activity of hDPSCs was assessed by alamarBlue™ assay, Fig. 3b showed that the metabolic activity of hDPSCs in both encapsulations were slightly increase at 24–96 h post encapsulation and tend to be stable until 336 h. Consistent results were visualized in Fig. 3c which encapsulated hDPSCs were stained by live/dead staining kit. All cells were stained by DAPI and the dead cells were stained by propidium iodide (PI) resulted in blue and red fluorescence, respectively. Only few numbers of dead cells were found at 336 h post-encapsulation.

### hDPSC characterization

**Fig. 4:**
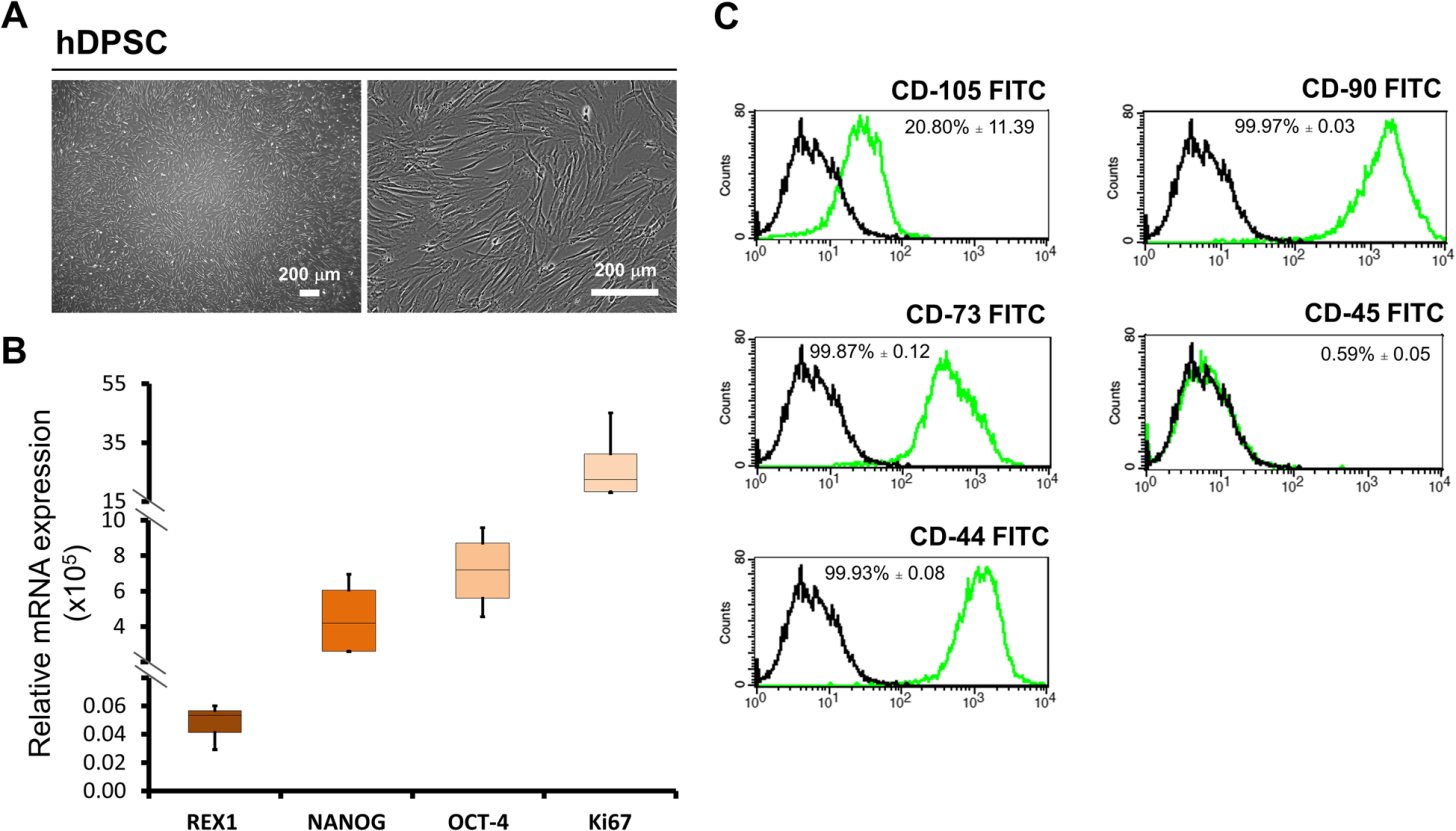
Morphology and characterization of hDPSCs. Morphological features of hDPSCs with low and high magnification were evaluated under light microscope **a**. The mRNA expression of stemness property genes (*REX1, NANOG* and *OCT4*) and proliferation gene (*Ki67*) were determined by RT-qPCR **b**. Expression of surface marker reflecting MSC property was also determined using flow cytometry **c**

hDPSCs were characterized before undergone IPC induction. Morphological assessment of hDPSCs under normal culture medium were performed. Plastic adherent and fibroblastic cells were observed from hDPSCs used in this experiment (Fig. 4a). In addition, RT-qPCR was employed to analyse the expression of stemness and proliferative genes. The results showed that the stemness genes (*REX1, NANOG* and *OCT4*) and proliferative gene (*Ki67*) were expressed by hDPSCs (Fig. 4b). The surface markers of hDPSCs were determined by using flow cytometry. Several mesenchymal stem cell surface markers CD90, CD73 and CD44 were strongly expressed. However, CD105 expression was relatively lower in hDPSCs. Furthermore, CD45 surface marker expression in hDPSCs were extremely low (Fig. 4c).

### In vitro differentiation of hDPSCs toward IPCs

**Fig. 5:**
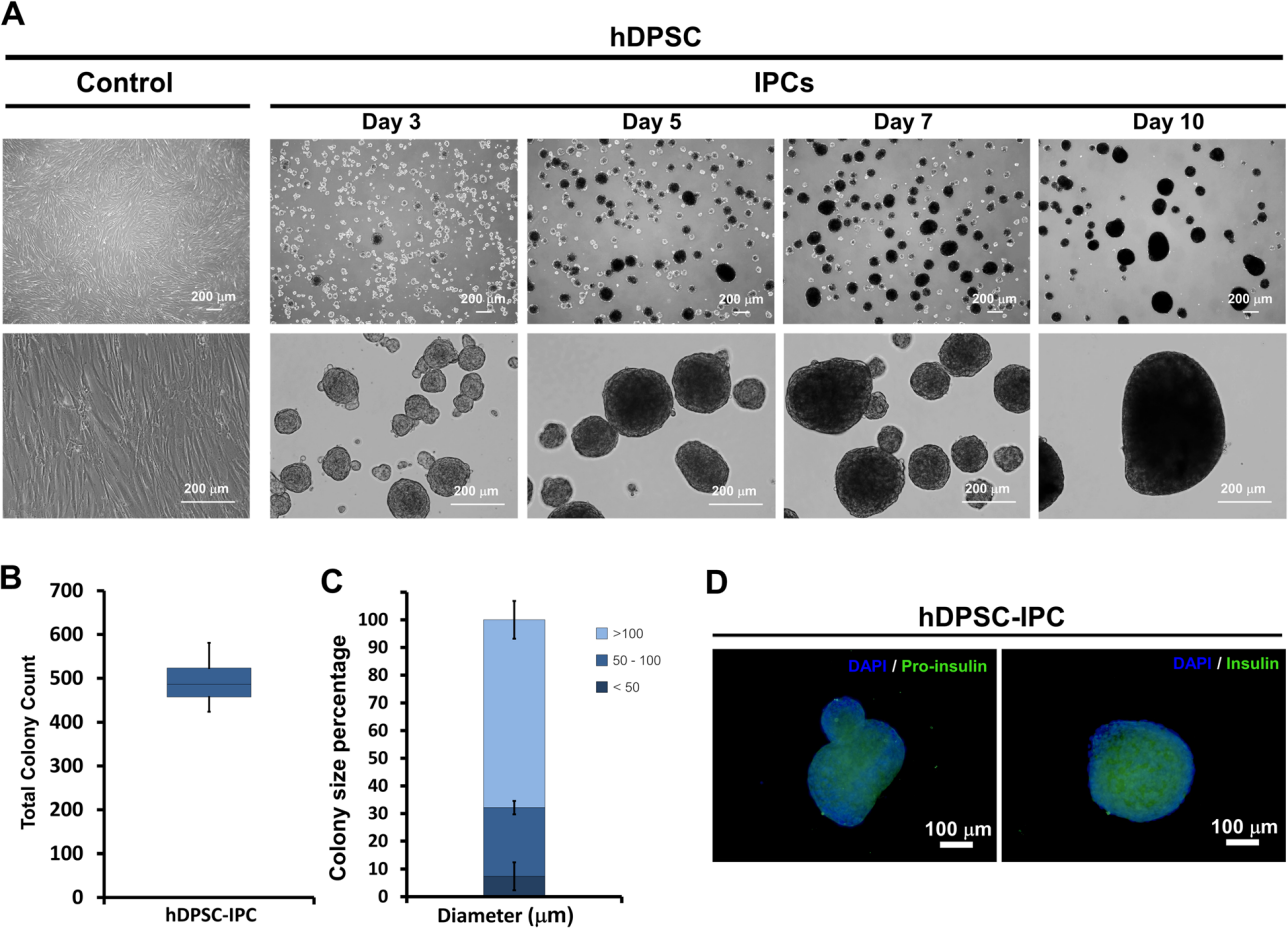
In vitro differentiation of hDPSCs toward IPCs. Different morphological appearances of hDPSCs after induction using three-stage differentiation protocol were illustrated at day 3, 5, 7 and 10 **a**. The total colony number **b** and the colony size distribution **c** were also investigated. The expression of pro-insulin and insulin were evaluated by immunocytochemistry staining **d**

Ten-day induction protocol was employed in this experiment. Initial differentiation was observed at day 3, where the single cell suspension of hDPSCs at day 0 were changed into cell aggregates. Further, the development of cell aggregates were clearly noted at day 5, day 7 and day 10. In the end of the induction protocol, big and dense cell aggregates were observed (Fig. 5a). The total colony count of cell aggregates obtained from this experiment were 424–581 colonies in which 60.68–74.70% had diameter more than 100 μm (100. 81–303. 43 μm), while small colonies (diameter less than 50 μm) were only 1.55–12.10% (Fig. 5b and c). The results were further analyzed by immunocytochemistry staining (Fig. 5d) and it was showed that cell aggregates derived from hDPSCs expressed both intracellular pro-insulin and insulin.

### In vitro viability evaluation of alginate/pluronic F127-based encapsulation of hDPSC-derived IPCs

**Fig. 6:**
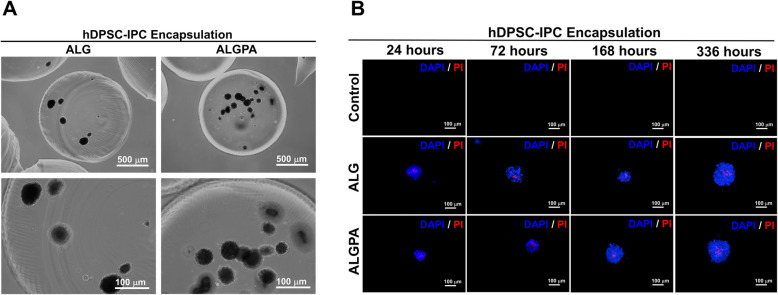
Morphology and viability evaluation of encapsulated hDPSC-derived IPCs using ALG and ALGPA. Encapsulated hDPSC-derived IPCs morphologies in both ALG and ALGPA were ilustrated **a**. The viability evaluation of encapsulated hDPSC-derived IPCs was also determined by live/dead staining **b**

Microscopic observation of encapsulated hDPSC-derived IPCs in both encapsulation platform was performed (Fig. 6a). The findings showed that cell aggregates derived from hDPSCs were unequally dispersed into the alginate beads. Cell aggregate damage was not observed. Further analysis to evaluate the viability of encapsulated hDPSC-derived IPCs was done by using live/dead staining (Fig. 6b). Post encapsulation staining showed that hDPSC- derived IPCs were survive at least 336 h. However, small mass of non-viable cells was observed in the core of encapsulated cell aggregates.

### Alginate/pluronic F127-based encapsulation maintains functionality of hDPSC-derived IPCs in vitro

**Fig. 7:**
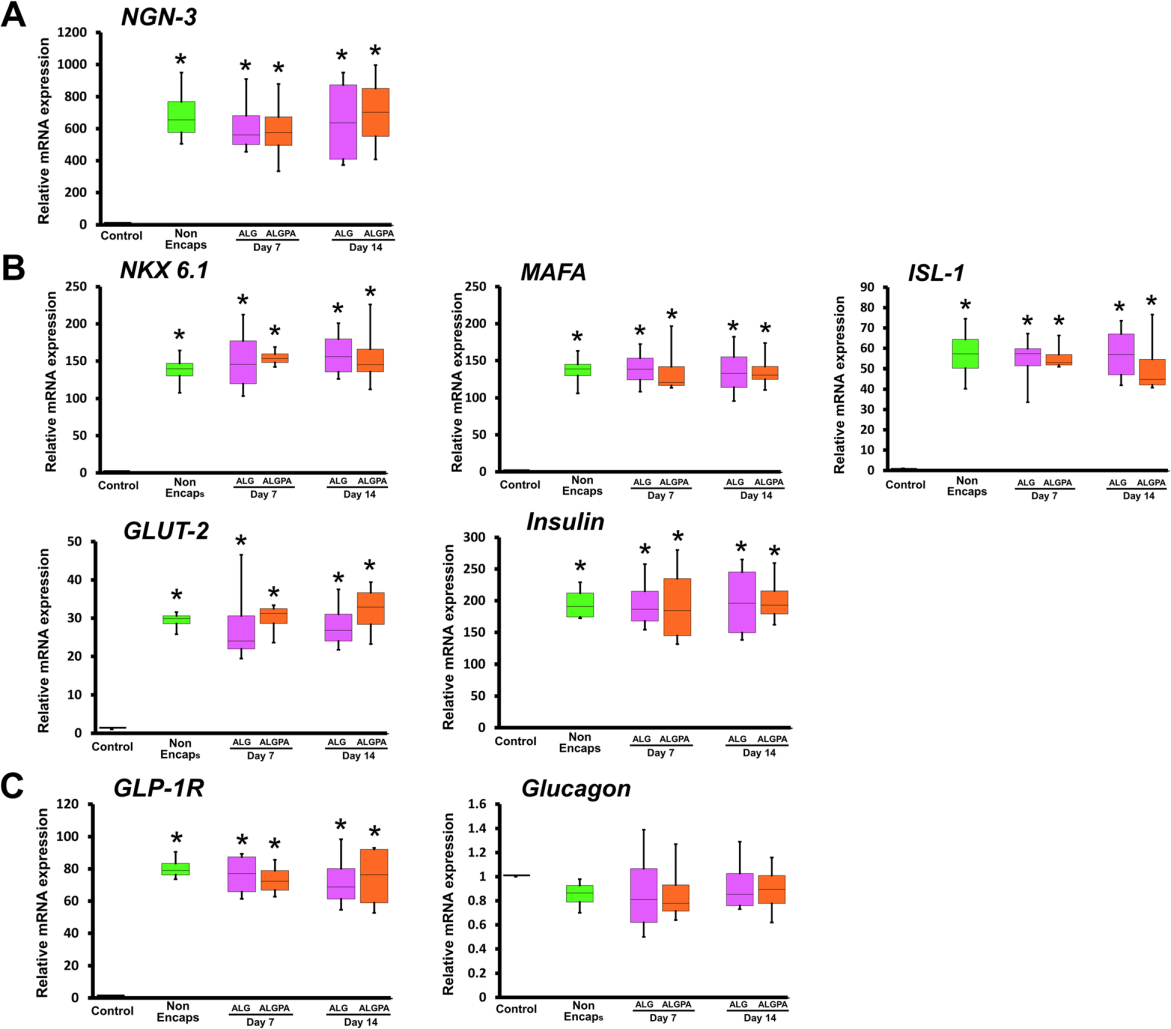
Pancreatic gene expression analysis of encapsulated hDPSC- derrived IPCs using ALG and ALGPA. The mRNA expression of pancreatic endoderm marker (*NGN3*) **a**, pancreatic islet markers (*NKX 6.1, MAFA, ISL-1, GLUT-2* and *INSULIN*) **b** and pancreatic related markers (*GLP-1R* and *GLUCAGON*) **c** by ALG- and ALGPA-encapsulated hDPSC-derived IPCs were determined by RT-qPCR at day 7 and 14 post encapsulation. The asterisks indicated significant difference, comparing with undifferentiated cell (*p* < 0.05)

Post-encapsulation evaluation of hDPSC-derived IPCs was performed by RT-qPCR to analyse the expression of pancreatic endoderm, pancreatic islet and pancreatic related genes. Encapsulated hDPSC-derived IPCs in ALG and ALGPA expressed pancreatic endoderm gene marker (*NGN3*) at day 7 and day 14 post encapsulation (Fig. 7a). The expression of pancreatic islet genes was noted at day 7 and 14. *NKX6.1, MAF-A, ISL-1, GLUT-2* and *INSULIN* genes were expressed in encapsulated hDPSC-derived IPCs in both ALG and ALGPA (Fig. 7b). Further, at day 7 and 14 in both encapsulation platform, the pancreatic-related gene, *GLP-1R,* was detected, while the expression of *GLUCAGON* gene was not significantly different compared to undifferentiated cells (Fig. 7c). All gene expression patterns regarding pancreatic endoderm, pancreatic islet and pancreatic-related genes of encapsulated hDPSC- derived IPCs were not significantly different compared to initial IPC condition before encapsulation.

**Fig. 8:**
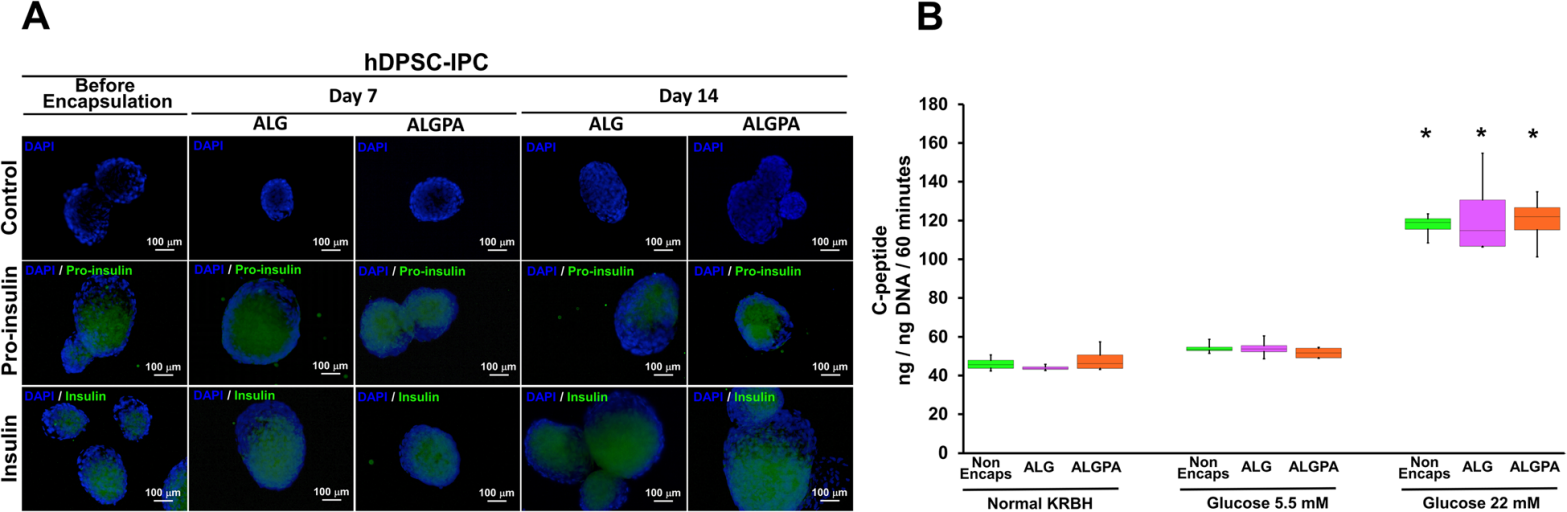
Pancreatic protein expression and functional analyses of encapsulated hDPSC-IPCs using ALG and ALGPA. The expression of pro-insulin and insulin by ALG- and ALGPA-encapsulated hDPSC-IPCs were evaluated by immunocytochemistry staining at day 7 and 14 post-encapsulation **a**. C-peptide secretion was also determined by glucose-stimulated C-peptide secretion (GSCS) assay **b**. The asterisks indicated significant difference, comparing with normal and glucose 5.5 mM in KRBH (*p* < 0.05)

Immunocytochemistry staining was employed to confirm the results. hDPSC-derived IPCs were stained before and after encapsulation (7 and 14 days). Figure 8a visualized that in both encapsulation by ALG and ALGPA, intracellular pro-insulin and insulin were expressed at day 7 and 14 post-encapsulation. The similar expression of intracellular pro-insulin and insulin also was observed in hDPSC-derived IPCs at the initial condition (before encapsulation).

In vitro evaluation regarding the function of encapsulated hDPSC-IPCs was assessed by using glucose-stimulated C-peptide secretion (GSCS) assay. The results of this experiments were compared with the initial condition of hDPSC-derived IPCs (before encapsulation). At day 14 post-encapsulation, hDPSC-derived IPCs in both ALG and ALGPA were challenged with three different condition, normal KRBH, 5.5 mM and 22 mM glucose in KRBH. The result showed that C-peptide was secreted by hDPSC-derived IPCs in both ALG and ALGPA encapsulation conditions after incubated with 22 mM glucose in KRBH. This result was significantly different compared to the C-peptide secreted in normal KRBH and 5.5 mM glucose in KRBH conditions, suggesting trend of glucose-responsive function. In this study, no significant difference was observed regarding the in vitro functional evaluation of hDPSC-derived IPCs before and after encapsulation (Fig. 8b).

## Discussion

The concept of cell encapsulation was first introduced almost nine decades ago, unfortunately, some clinical trials regarding the application of cell encapsulation have not led any approval for clinical application [24]. Currently, stem cell-based diabetes therapy offers promising strategy for T1DM and eliminate the obstacles of Edmonton protocols [25]. Encapsulation platform can be served to provide viable allogeneic or xenogeneic cells for its purpose [24].

In this study, both ALG and ALGPA encapsulations were validated. The encapsulation method in this study was based on the manual nozzle extrusion as hDPSC-derived IPCs were extruded through needle as a nozzle tip. The size of needle plays an important role since the suitable needle size should be fulfilled to avoid the cell aggregate damage [26]. In present study, needle size 22G with the inner diameter 0.413 mm was chosen because it provided suitable inner diameter for generated hDPSC-derived IPC size. Permeability is one of important factor for cell encapsulation application [27]. The ALG and ALGPA permeability against glucose and insulin was reported in this study. Similar studies demonstrated the permeability of alginate using mouse insulinoma and glucose-responsive rat cell line. The results of these studies reported that encapsulation of both cell lines could keep the ability of insulin secretion in response to extra-capsular glucose stimulation [28, 29]. Gautier et al. (2011) reported that glucose, ammonia, vitamin B12 and another low to middle molecular weight substance can easily diffuse across the alginate [30]. The results regarding bead swelling assay showed that, both encapsulation by ALG and ALGPA were relatively stable. Assessment of bead swelling was aimed to determine the stability of the beads. In some conditions, alginate beads can swell resulted in the increasing of porosity and bead damage [31]. The swelling behaviour can occur mainly due to the osmotic factors. In PBS, high concentration of Na^+^ can cause bead swelling, but in some studies, alginate beads showed the elasticity without showing membrane breakage [31]. In this experiment, high purity of alginate was employed to avoid over-swelling. The substances (i.e. proteins or endotoxins) that could increase the chemical potential of the solvent (negative charge) inside the capsule were not detected. Capsule deformation can occur by the rapid change of the environment. In consequence, the adaptation to a new environment is occurred to achieved the equilibrium of chemical potential of the solvent inside and outside the capsule [32]. In this study, pore morphology was not reported, however similar study with the same alginate type and ionic crosslink solution showed that concentration of 0.5–1% alginate solution could produce a bead with pore diameter approximately 7.2–8.0 nm which inhibited 21–25 kDa of dextran and 78–103 kDa of protein including immunoglobulin G [33]. In addition, the pore size of the beads can be reduced by increasing the alginate concentration [34].

In the present study, biocompatibility of both encapsulation ALG and ALGPA was assessed. The alamarBlue™ assay, a redox indicator, was employed in this study. In three-dimensional (3D) culture system using alginate matrix, choosing the most reliable and precise assay to assess the viability plays an important role. Tetrazolium salt-based assay is widely used to evaluate the cell viability in two-dimensional (2D) culture system, however the highly toxic of DMSO or HCl/isopropanol used in the assay led an obstacle [35]. Moreover, multiple metabolic reactions in both cytoplasm and mitochondria can be assessed by alamarBlue™ assay. This assay is based on the oxidation reduction caused by nicotanimide adenine dinucleotide phosphate hydrogen (NADPH), flavine adenine dinucleotide hydrogen (FADH_2_), flavine adenine mononucleotide hydrogen (FMNH_2_), nicotinamide adenine dinucleotide hydrogen (NADH), cytochromes and all cellular respiration metabolic reaction [36]. During the first 48 h post-encapsulation, the metabolic activity of hDPSCs was slightly increase. It might cause by the absorption and ion exchange was slow due to the encapsulation compare with 2D culture system. Cell-to-cell communication was also limited by this platform, consequently the cells need longer time for acclimatization [37]. Further, after 96 h post-encapsulation the metabolic activity of hDPSCs was relatively stable. The evidence from another study showed that alginate encapsulation limited the cell proliferation and remained the cell into G0 stage [38]. Moreover, qualitative observation incorporating live/dead staining visualized similar condition. More numerous viable cells were observed more visible compared with non-viable cells after 96 h post-encapsulation.

Based on The International Society for Cellular Therapy, the minimum criteria of MSCs were established in 2005. The criteria that should be fulfilled for MSCs included showing fibroblastoid in morphology, adhering in culture plate/flasks under normal culture condition and exhibiting CD73, CD44, CD90 and CD105 surface markers, whereas lacking express of CD45 marker [39]. These minimum criteria were fulfilled by the hDPSCs used in this study. The results of present study were consistent as previous studies that all of MSCs surface markers (CD73, CD44, CD90 and CD105) could be detected in hDPSCs [13, 40]. In addition, pluripotency transcription factors *REX1, NANOG* and *OCT4* were expressed, which was in agreement with previous study conducted by Shivakumar et al. (2019) [41].

Currently, various transdifferentiation protocols of IPCs were reported. Non integrative methods are widely used in MSC-based differentiation protocol, in comparison with integrative methods, where foreign sequences were transduced into host genome are mainly used in induced pluripotent stem cells (iPSCs) [42]. Further, the risk of tumor formation and gene alteration still become the concern regarding safety aspect in clinical application [43]. However, several studies reported the safety aspect of adenoviral and Sendai viral integrative methods [44]. In the present study, non-integrative method using small molecules and peptides was employed for IPC induction. In MSC-based protocols, two main step IPC differentiation were involved. The initial stage, MSCs were induced into pancreatic progenitor followed by β-cell maturation [45]. However, three-stage differentiation protocol was involved in this study. The initial stage of IPC differentiation was the induction of hDPSCs into definitive endoderm incorporated the combining activin A and sodium butyrate. A pioneer study reported the successful of the murine adipose-derived stem cells (ASCs) induction into definitive endoderm by using these combinations of small molecules. The results was confirmed by the expression of definitive endoderm protein markers SOX17, Foxa2, HNF-1β, also gene markers *Foxa2, CK-19 and GATA-4* [46]. Definitive endoderm differentiation also was achieved in hESCs after activin A treatment [47]. L-taurine was employed in the pancreatic endoderm differentiation stage. Similar substance was used for murine ASC differentiation toward pancreatic endoderm [46] and human placenta-derived mesenchymal stem cells (hPDMSCs) [48]. In murine ASC study incorporated physiological dose of L-Taurine, the gene expression of *PDX-1, NGN3, NeuroD, Pax4 and NKX2.2* was achieved in day 5 post induction [46]. In this study, *NGN3* expression was noted at day 5. Final step of β-cell maturation was achieved by adding combination of small molecules and peptides i.e. high dose of L-taurine, nicotinamide and glucagon-like peptide (GLP)-1. The β-cell-related markers, *NKX6.1, MAFA, ISL-1, GLUT-2* and *INSULIN* were expressed in hDPSC-derived IPCs. *PDX1* and *NKX6.1* are important transcription factors for β-cell maturation [49]. In this study, the matured and functional β-cell still could be achieved in the absence of *PDX1* expression, it might caused by the expression of *NKX6.1* that could maintain *MAFA* as a transcription factor for insulin gene expression [44]. Additionally, *GLP-1* was reported to increase and stabilize the expression of *INSULIN* mRNA, subsequently the secretion and stimulation of insulin was enhanced [44, 46].

In the present study, qualitative in vitro viability assessment of encapsulated hDPSC-derived IPCs in ALG and ALGPA showed that both encapsulation platform could provide suitable environment for hDPSC-derived IPCs. The essential requirements for cell culture i.e. porosity, stability and permeability were fulfilled by alginate [50]. Consistent results by several studies were reported that pancreatic islet, ESC- and iPSC-derived IPC encapsulation using alginate could maintain the viability both in vivo and in vitro [51–56]. Another study on pancreatic islet cryopreservation incorporated alginate encapsulation reported that alginate encapsulation could maintain both viability and functionality of pancreatic islets [57]. According to the functionality evaluation of hDPSC-derived IPC encapsulation in ALG and ALGPA, the results of this study showed that this encapsulation platform could maintain the expression of pancreatic endoderm and pancreatic β-cell gene markers at least for 14 days. In undifferentiated ESC and iPSC encapsulation study, post-encapsulation induction in alginate could enhance both gene and protein expression of mature β-cell markers (*PDX1, MAFA* and *INSULIN*) compared to induction in tissue culture plastic (2D system) [51, 54]. However, in MSC-derived IPC study using trabecular meshwork-derived MSCs (TM-MSCs), the mature and functional IPCs in tissue culture plates and alginate microfiber induction did not show significant difference [50]. In addition, encapsulation of differentiated cells (mature β-cell) was remained difficulties. Study in hESC-derived IPCs, showed that mature β-cell encapsulation viability still could be maintained, but some mature phenotype (insulin secretion) was slightly decreased [54]. Post-encapsulation induction of undifferentiated hESCs resulted in adequate mature β-cell. However, in vitro differentiation of post-encapsulated hESCs seemed impractical for clinical application since adequate amount of mature β-cell was difficult to achieve in static culture system [54]. Moreover, the contact of undifferentiated cells under induction media should be minimized to avoid the antigen contamination from dead cells [51]. Beside the beneficial aspect of encapsulation in providing immunoisolation properties, currently, the cell encapsulation technology has been used to treat some diseases i.e. cardiac disease, hepatic disease and bone impairment [58–63] by using several biomaterials i.e. gelatin methacrylate [58], poly (3-hydroxybutyrate) [59], sodium alginate, poly (ethylene glycol) [61], poly (lactic-co-glycolic acid) [63] and poly(N-isopropylacrylamide-co-acrylic acid) or P (NIPAM-AA) [60]. To date, commercially available and FDA approved of encapsulation technology to cure type I DM is not available. Some private companies i.e. Living Cell Technology (DIABECELL**®**), Sernova Corp. (Their Cell Pouch System™), ViaCyte (PEC-Encap™) and Beta-O_2_-Technology (β-Air**®** device) are still conducting US Phase I/II clinical studies. Unfortunately, among these technologies, MSC-derived IPCs have not been reported to use as the cell source. They are using xenogenic porcine-derived islets, allo-islets, hESC-derived pancreatic endocrine cells and iPSC-derived IPCs respectively [64]. This present study demonstrated for the first time regarding the mature MSC-derived IPC encapsulation. In both encapsulation by ALG and ALGPA, intracellular pro-insulin and insulin expression were detected at day 7 and 14 post-encapsulation. These results were consistent with the pancreatic endoderm and pancreatic islet marker gene expression maintained by ALG and ALGPA encapsulation. Finally, the functional property of encapsulated hDPSC-derived IPCs in both ALG and ALGPA was confirmed by the ability of glucose-responsive C-peptide secretion.

## Conclusion

In summary, this study is firstly demonstrating the hDPSC-derived IPC encapsulation in alginate and alginate/pluronic F127. The results from this study suggested that, in both encapsulation platform, the viability and functionality of hDPSC-IPCs could be maintained. However, the role of pluronic F127 in this study was not clearly shown. Further, in vivo study needs to be conducted to evaluate the role of pluronic F127 regarding the ability to enhance angiogenesis, to avoid extra capsular cellular overgrowth and related factors for successful of transplantation.

## Materials and methods

### hDPSC isolation and culture

Human DPSCs were isolated from human dental pulp tissues of extracted premolars and molars according to wisdom teeth issues under patients’ informed consents and ethical approval from the Human Research Ethic Committee, Faculty of Dentistry, Chulalongkorn University (HREC-DCU 2018/054). Tissue explant technique was used, based on previous protocol [13]. Cells were seeded and maintained in high glucose Dulbecco’s Modified Eagle Medium (DMEM; Thermo Fisher Scientific Corporation, USA) with supplementation of 1% of Antibiotic-Antimycotic (Thermo Fisher Scientific Corporation, USA), 1% GlutaMAX™ (Thermo Fisher Scientific Corporation, USA), and 10% fetal bovine serum (FBS) (Thermo Fisher Scientific Corporation, USA) under 37 °C in humidified environment with 5% CO_2_ condition. Culture medium was changed every 48 h. Cells were subculture when 80% confluence reached. Four different cell lines in passage 2–5 were used in the experiments.

### hDPSC characterization

hDPSCs were characterized according cell morphology, stemness and proliferative mRNA marker expression, and surface marker analysis. Cell morphology was captured by phase-contrast microscope. RT-qPCR was used to analysed mRNA marker expression regarding stemness property (*REX1, NANOG*, and *OCT4*) and proliferative marker (*Ki67*). Cells were then characterized by flow cytometry for MSC surface markers. Briefly, the cells were stained with FITC-conjugated anti-human CD105^+^ antibody (Bio Legend, California, USA), FITC-conjugated anti-human CD73^+^ (Ecto-5′-nucleotidase) antibody (Bio Legend, California, USA), FITC-conjugated anti-human CD90^+^ (Thy1) antibody (Bio Legend, California, USA), FITC-conjugated anti-human CD44^+^ antibody (Bio Legend, California, USA), and FITC-conjugated anti-human CD45^−^ antibody (Bio Legend, California, USA). FITC-conjugated Mouse IgG1κ isotype control (FC) antibody (Bio Legend, California, USA) was used as an isotype control for this assay. The assay was performed by using FACScallibur flow cytometer with CellQuest software (BD Bioscience, New Jersey, USA).

### In vitro differentiation of IPCs

IPC differentiation was performed by using 10 days 3-step differentiation protocol as performed by Sawangmake et al. (2014) (13). Briefly, 1 × 10^6^ of hDPSCs were seeded in 60 mm non-treated culture dish (Eppendorf, Hamburg, Germany) as a single cell suspension. The cells were maintained in serum-free medium (SFM)-A for 3 days, SFM-B for 2 days and SFM-C for 5 days and the medium were changed every 48 h. The medium used in this experiment were SFM-DMEM (Thermo Fisher Scientific Corporation, USA) with different supplementation. The supplementation of each medium were respectively as follows; SFM-A: 1% BSA (Cohn fraction V, fatty acid free; Sigma, Missouri, USA), 1X insulin-transferrin-selenium (ITS) (Invitrogen, USA), 4 nM activin A (Sigma, Sigma, Missouri, USA), 1 nM sodium butyrate (Sigma, Missouri, USA), and 50 μM beta-mercaptoethanol (Sigma, Missouri, USA); SFM-B: 1% BSA, 1X ITS, and 0.3 mM taurine (Sigma, Missouri, USA); and SFM C: 1.5% BSA, 1X ITS, 3 mM taurine, 100 nM glucagon-like peptide (GLP)-1 (Sigma, Missouri, USA), 1 mM nicotinamide (Sigma, Missouri, USA), and 1x non-essential amino acids (NEAAs) (Sigma, Missouri, USA).

### Alginate and alginate/pluronic F127 bead fabrication and morphological observation

Alginate bead fabrication was performed by manual extruding method using plastic syringe with different needle size (22G, 24G and 26G). In brief, 2.0% (w/v) sodium alginate (Sigma, Missouri, USA) solution was extruded into 100 mM CaCl_2_ gelling solution for 5 min. The crosslinked process was performed under stirring condition to avoid beads coalescence during polymerization. The beads were removed and were washed in Kreb-Ringer HEPES (KRH) buffer (pH 7.22) containing 2.5 mM CaCl_2_, 132 mM NaCl, 4.7 mM KCl, 1.2 mM MgCl_2_.6H_2_O, 25 mM HEPES and 2.52 CaCl_2_.2H_2_O for 5 min under stirring condition. For double coating, the alginate beads were incubated in 30% (w/v) pluronic F127 (Sigma, Missouri, USA) solution for 3 min at room temperature. Pluronic F127 coated beads subsequently wash 3 times by using phosphate-buffered saline (PBS) pH 7.4. All the protocols were performed under sterile condition. Generated beads were maintained in PBS under 37 °C in humidified environment with 5% CO_2_ condition for 28 days. Morphological evaluation was performed under inverted microscope (EVOS – Invitrogen, California, USA). The beads diameter was analysed by using Image J software (National Institute of Health, Maryland, USA).

### Modified-transwell diffusion study

Costar brand (Corning, New York, USA) 24-well 8 μm-pore size transwells were used in this study. Transwell insert barrier were modified by coating using 1.5 mm alginate and/or 1.0 mm pluronic F127 in thickness. Transwell insert barriers were assembled into respective well, subsequently 100 μL of 22.2 mM glucose solution or insulin standard solution were filled in the upper compartment while 600 μL PBS in the lower compartment. The transwell culture plate were maintain in 37 °C incubator for diffusion study. After 5, 15, 30, and 60 min-incubation, the solution in the lower compartments were collected for glucose quantification using glucose liquocolor GOD-PAP method (Human, Wiesbaden, Germany) and insulin level quantification after 30 and 60 min incubation by using human Insulin ELISA kit (EMD Millipore, Burlington, Massachusetts, USA).

### hDPSC and hDPSC-derived IPC encapsulation

Single cell hDPSCs at a concentration of 1 × 10^6^ cell/mL and cell aggregates of hDPSC-derived IPCs from previous protocol were resuspended in 2.0% alginate solution. The encapsulation of hDPSCs and hDPSC-derived IPCs were performed by using manual extruding method as described in previous part. Subsequentially after encapsulation protocol, the beads were removed and maintained in normal medium for encapsulated hDPSCs and SFM-C for encapsulated hDPSC-derived IPCs under 37 °C in humidified environment with 5% CO_2_ condition. The culture medium will be changed every 48 h until the following analyses.

### Cell viability assay

Encapsulated hDPSCs and hDPSC-derived IPCs were evaluated for their viability using qualitative live/dead fluorescent staining kit, The NUCLEAR-ID® Blue/Red cell viability reagent (GFP-CERTIFIED®) (Enzo Life Science, Farmingdale, New York, USA), according to the manufacture protocol. The results were interpreted using fluorescent microscope equipped with Carl Zeiss™ Apoptome.2 apparatus (Carl Zeiss, California, USA). Furthermore, the alamarBlue™ (Invitrogen, California, USA) were used to evaluate the encapsulated hDPSCs. In brief, encapsulated hDPSCs were maintained in normal medium containing 10% (v/v) alamarBlue™ for 20 h. To determine the percent reduction of alamarBlue™, the solution was measured using spectrophotometer at 570 and 600 nm wavelength.

### Capsule dissolution

Prior to post-encapsulation evaluation, ALG and ALGPA encapsulated hDPSC-derived IPCs were dissolved by incubating in dissolving buffer (0.1 M EDTA and 0.2 M C_6_H_5_Na_2_O_7_.2H_2_O, pH 7.4) for 5 min in 37 °C. hDPSC-derived IPCs from degraded alginate beads were washed in PBS three times 3 min each, and processed for further analysis (RT-qPCR and immunocytochemistry staining).

### Reverse transcription-quantitative real time polymerase chain reaction (RT-qPCR)

TRIzol™ reagent (Invitrogen, Callifornia, USA) was used for cellular RNA extraction, subsequentially the cDNA was obtained from 1 μg RNA using reverse transcriptase enzyme kit (Promega, Wisconsin, USA). Reverse transcription-quantitative real time PCR (RT-qPCR) using FastStart® Essential DNA Green Master® (Roche Diagnostic, Risch-Rotkreuz, Switzerland) was performed to detect the gene expression by CFX96™ real time PCR detection system (Bio-Rad, California, USA). The mRNA value will be presented as relative mRNA expression by normalized to 18S ribosomal RNA and the control. The formula 2^-∆∆Ct^ will be used to calculate normalization and fold change. The primer sequences were shown in Table 1**.**

**Table 1:** List of Primers

Genes	Accession number	Forward Primer Reverse Primer	Length (bp)	Tm(°C)
*NANOG*	NM_024865.4	5′ – ATGCCTCACACGGAGACTGT – 3′ 5′ – AAGTGGGTTGTTTGCCTTTG – 3′	103	61.19 57.31
*OCT-4*	NM_002701.6	5′ – TCGAGAACCGAGTGAGAGG – 3′ 5′ – GAACCACACTCGGACCACA – 3′	125	58.14 59.56
*REX-1*	NM_174900.5	5′ – TGGGAAAGCGTTCGTTGAGA − 3′ 5′ – CACCCTTCAAAAGTGCACCG – 3′	90	59.89 59.97
*Ki67*	NM_001145966.1	5′ – TCAGAATGGAAGGAAGTCAACTG – 3′ 5′ – TCACTCTCATCAGGGTCAGAAG – 3’	105	58.35 58.90
*PDX-1*	NM_000209.4	5’ – AAGCTCACGCGTGGAAAGG – 3′ 5′ – GGCCGTGAGATGTACTTGTTG – 3’	145	57.89 52.38
*NGN-3*	NM_020999.3	5’ – CGGTAGAAAGGATGACGCCT – 3′ 5′ – GGTCACTTCGTCTTCCGAGG – 3’	138	59.54 60.11
*NKX-6.1*	NM_006168.2	5’ – TTGGCCTATTCGTTGGGGAT – 3′ 5′ – GTCTCCGAGTCCTGCTTCTTC – 3’	125	59.08 60.14
*MAFA*	NM_201589.3	5’ – GCACATTCTGGAGAGCGAGA – 3′ 5′ – TTCTCCTTGTACAGGTCCCG – 3’	102	59.83 58.74
*ISL-1*	NM_002202.2	5’ – TCCCTATGTGTTGGTTGCGG - 3′ 5′ – TTGGCGCATTTGATCCCGTA – 3’	200	60.32 60.39
*GLUT-2*	NM_000340.1	5’ – GGTTTGTAACTTATGCCTAAG – 3′ 5′ – GCCTAGTTATGCATTGCAG – 3’	211	52.25 54.24
*INSULIN*	NM_000207.2	5’ – CCGCAGCCTTTGTGAACCAACA – 3′ 5′ – TTCCACAATGCCACGCTTCTGC – 3’	215	64.34 64.45
*GLP-1R*	NM_002062.4	5’ – TCGCTGTGAAAATGAGGAGGA – 3′ 5′ – TCACTCCCGCTCTGTGTTTG – 3’	189	59.38 60.25
*GLUCAGON*	NM_002054.4	5’ – TTATTTGGAAGGCCAAGCTGC – 3′ 5′ – GTCTGCGGCCAAGTTCTTCA – 3’	110	59.45 60.88
*18S*	NR_003286.2	5’ – GTGATGCCCTTAGATGTCC – 3′ 5′ – CCATCCAATCGGTAGTAGC – 3’	233	55.04 54.86

### Immunocytochemistry staining

Cell colonies of hDPSC-derived IPCs were fixed in cold methanol, subsequently 0.1% Triton®-X100 (Sigma, Missouri, USA) in PBS to permeabilized the sample, and then were incubated with 10% donkey serum in PBS for 1 h for background reducing. The primary antibodies used in this experiment were mouse anti-human pro-insulin (EMD Millipore, Burlington, Massachusetts, USA) and mouse anti-human insulin (EMD Millipore, Burlington, Massachusetts, USA) at the dilution 1:100. FITC-conjugated goat anti-mouse IgG (Abcam, Cambridge, United Kingdom) at dilution 1:1000 were used as secondary antibody. DAPI (0.1 μg/mL) was used to counterstain the nucleus. Interpretation of this assay were performed by analyzing under fluorescent microscope equipped with Carl Zeiss™ Apoptome.2 apparatus (Carl Zeiss, California, USA).

### Glucose stimulated C-peptide secretion (GSCS) assay

Glucose-stimulated C-peptide secretion (GSCS) assay was performed at day 14 post encapsulation by incubating encapsulated hDPSC-derived IPCs in normal KRH NaHCO_3_ (KRBH) pH 7.22 (120 mM NaCl, 5 mM KCl, 2.5 mM CaCl_2_.2H_2_O, 1.1 mM MgCl_2_.6H_2_O and 25 mM NaHCO_3_), 5.5 mM of glucose anhydrous (Sigma, Missouri, USA) in KRBH, or 22 mM glucose anhydrous (Sigma, Missouri, USA) in KRBH for 60 min in each buffer solution. Enzyme-linked immunosorbent assay (ELISA) was used to quantified C-peptide level by using human C-peptide ELISA kit (EMD Millipore, Burlington, Massachusetts, USA) based on manufacturing protocol. Subsequently, encapsulated hDPSC-derived IPCs were disolved using dissolving buffer, cell collony were collected and DNA extraction was perform by using DNeasy Blood and Tissue Kits (Qiagen, Venlo, Netherlands). The level of C-peptide was normalized with the total DNA (ng) and stimulation time (minutes).

### Statistical analysis

The results of this study were presented as box plot with whisker bar. Non-parametric statistical analysis and four replicates for each cell type (*n* = 4) were used in the study. The statistical analysis was performed by using SPSS Statistics. To compare two independent groups, the Mann Whitney U test was employed, while Kruskal Wallis test and pairwise comparison were used for three or more group comparison. Statistically significant difference was recognized when *p*-value < 0.05.

## Data Availability

The datasets used and/or analyzed during the current study are available from the corresponding author on reasonable request.

## Abbreviations

IPC: Insulin-producing cell
hDPSC: Human dental pulp stem cell
ESCs: Embryonic stem cells
MSCs: Mesenchymal stem cells
BM-MSCs: Bone marrow-derived mesenchymal stem cells
SHED: Stem cells from human exfoliated deciduous teeth
RT-qPCR: Real time quantitative polymerase chain reaction
FITC: Fluorescein isothiocyanate
NADPH: Nicotanimide adenine dinucleotide phosphate hydrogen
FADH_2_: Flavine adenine dinucleotide hydrogen
FMNH_2_: Flavine adenine mononucleotide hydrogen
NADH: Nicotinamide adenine dinucleotide hydrogen
iPSCs: Induced pluripotent stem cells
ASCs: Adipose-derived stem cells
hPDMSCs: Human placenta-derived mesenchymal stem cells
TM-MSCs: Trabecular meshwork-derived mesenchymal stem cells
